# The Time Has Come; One Year Has Passed

**Published:** 2013

**Authors:** Fatemeh Heidary, Abolfazl Rahimi, Reza Gharebaghi

**Affiliations:** 1 School of Medicine, Shahid Beheshti University of Medical Sciences, Tehran, Iran; 2 Dept. of Ophthalmology, Tehran Medical Branch, Islamic Azad University, Tehran, Iran; 3 Editorial Office, Medical Hypothesis, Discovery & Innovation Ophthalmology Journal

**Keywords:** Ophthalmology, Medical Hypotheses, Idea, Future strategy

Few days ago our advisory editorial board member Prof. Gholam A. Peyman was named as one of the 12 eminent researcher recipients of the National Medal of Technology and Innovation in US. Eleven extraordinary inventors were also named recipients of the National Medal of Technology and Innovation. This award and the National Medal of Science designations are the uppermost honors bestowed by the federal government upon scientists, engineers, and inventors. On behalf of editorial board we would like to congratulate Prof. Peyman for his great achievement.

## Importance of hypotheses

Unless additional ophthalmic services are made available universally, the number of people suffering from vision loss due to chronic ocular diseases would rise as a result of increased life expectancy and population growth thus new innovation and discoveries are requires.

Hypotheses originate from real data, and drive the development of new data [1]. On the first birthday of our Medical Hypothesis, Discovery & Innovation (MEHDI) Ophthalmology Journal, it would be our pleasure to quote a paragraph from Prof David F Horrobin on importance of hypotheses in medical sciences when he founded Medical Hypotheses Journal in 1975: 

“Scientific progress depends on the existence of creative tension between ideas and observations. An observation is made which cries out for explanation. A hypothesis is proposed to account for the observation. The hypothesis is tested by making more observations which almost invariably require the abandonment of some part of the hypothesis. The physical and chemical sciences long ago recognized that observations are not superior to hypotheses in generating scientific progress nor are hypotheses superior to observations. Both are necessary [2]”.

## Happy birthday!

In fact, with globalization fast approaching, those of us in visual sciences must be able to quickly respond in this era of ongoing swift and sweeping change. During 2012 we received overwhelmingly positive endorsement from our readers, reviewers, and editorial board members to continue the work on our journal although we faced with financial difficulties. 

After one year of publication with four regular issues, we received considerable feedback from around the world and gained many new colleagues. 

At this time, we are asking our readers to reply to a short and simple survey. The purpose of this survey is to identify our readers’ needs. The results would be used to help drive content and make improvements in future issues. We ask for responses from the readers, reviewers and editorial team to the following questions:

1. What do you hope to see in our journal?

2. What would encourage you to participate (e.g. submit an article or to become an author, reviewer or editorial board member)?

3. How do you envision using this journal?

4. How would new ideas contribute to the successful operation of this journal?

The answerers would be analyzed by our statistical consultant and the best responses would receive USD 700 award and an appreciation letter. The award would be a coupon for free advanced editing services to manuscripts. It is estimated that this money would subsidize the editing of approximately two manuscripts or 12 abstracts.

## Future strategies

We anticipate our journal evolving over time as we update features to meet readers’ needs. Manuscripts, participation, and insights will shape the future of this journal. We recommend that authors explain ideas and hypotheses clearly and coherently and include guidelines for testing their ideas. We are not looking for detailed responses to this question; general explanations will suffice. This journal intends to publish papers that offer solutions in the context of individual socioeconomic status, focusing on those in the lower financial strata as well describes in former editorials.

One of the areas of great interest to us is the current controversy in ophthalmology regarding the many treatments and studies in visual sciences. As of yet, no consensus has been reached and we are planning to publish scientific papers in 2013 on the subject. 

It is our contention that MEHDI Ophthalmology Journal will definitely be one of the journals that has the potential to engender collaborative research publication with similar journals such as Medical Hypotheses, Medical Hypotheses and Research (MHR), and Journal of Medical Hypotheses and Ideas. Ideally, at some point in the not too distant future, all journals will work together to provide a forum for developing thoughts and publishing hypotheses and become a cohesive unit for constructive interactions. In general, a future collaboration of this nature would seem to be ideal. 

We cannot forget when an Asian researcher who was naturally innovative minded wrote us with awe that in the current issue of ophthalmology journal a study had been performed the idea of it had stricken him 20 years before. Another PhD candidate said that he had a hypothesis in mind but found out later that the same idea had been published in another part of the world. Needless to say, that potential author regretted not having his idea patented in his own name. Therefore, this is of great benefit to those of you looking to publish: by making your hypotheses public, you are avoiding the potential trap of finding your idea or study in a current issue of another ophthalmology journal.

A philosopher of science puts hypotheses alongside experimentation as the correct scientific method [3]. Given that science continues to progress, we should move in tandem with the science. Our journal is enthusiastic about presenting information that is important, reliable and valid. Ideas that can correlate between basic sciences and clinical sciences should decrease the cost of healthcare services. Theoretically, they could be practiced in industry while making a contribution to modern science. Documentation of efforts such as these will be enthusiastically published.

We would appreciate readers in choosing this issue; a medium where information can be made available to all ophthalmology professionals very quickly. In this issue and in the editorial section of upcoming issues, we will discuss the possible short term strategies of the journal and look forward to reviewers or authors continuing to improve us. 

The last but not least, the MEHDI ophthalmology journal continue to play a constructive role in upholding focused on becoming a peer-reviewed, rigorous, and open-minded forum for new ideas and hypotheses in the field of visual sciences. We wish adhere to multilateralism and welcome your participation in this endeavor.
